# The Dislocated Hip Hemiarthroplasty: Current Concepts of Etiological factors and Management

**DOI:** 10.2174/1874325001711011200

**Published:** 2017-10-31

**Authors:** Carl Jones, Nikolai Briffa, Joshua Jacob, Richard Hargrove

**Affiliations:** 1 SpR in Trauma and Orthopaedics Waikato Hospital, Pembroke Street, Hamilton, New Zealand; 2 Frimley Park Hospital, Portsmouth Road, Frimley, Surrey, United Kingdom St Georges Hospital, Blackshaw Road, Tooting, SW17 0QT, London, UK

**Keywords:** Hip dislocation, Hip fracture, Hip hemiarthroplasty dislocation, Intracapsular neck, Dislocation, Epidemiology

## Abstract

**Background:**

Hip hemiarthroplasty (HA) following an intracapsular neck of femur fracture is an increasingly common procedure as a result of an ageing population. Patients are often frail and so morbidity and mortality figures are significant. As a result the National Institute for Health and Clinical Excellence (NICE) has formulated guidelines and a Best Practice Tariff (BPT) in an attempt to improve the care of such patients. Dislocation following HA is a potentially devastating complication with a reported incidence ranging from 1 to 15%. Multiple causative factors have been cited and studied in an effort to reduce the incidence of this complication which has a high rate of recurrence following the first episode and is associated with a high mortality rate and significant financial burden on the health economy. This paper reviews the available literature in an effort to identify the most pertinent factors affecting dislocation rates and thus reduce the incidence of this serious complication.

**Methods:**

A comprehensive review of the literature was performed using the search engine PubMed with the keywords ‘hip’, ‘hemiarthroplasty’ and ‘dislocation’. Two hundred and forty three articles were identified and assessed by the 3 authors independently. Data from fifty-two articles pertinent to the review on hemiarthroplasty dislocation epidemiology, risk factors and management were extracted in a standardised fashion.

**Results:**

Following review of the papers multiple causative factors relating to HA dislocation were identified and grouped into 4 broad categories for analysis. The factors with the strongest correlation with dislocation included patient cognition, previous failed surgery, delay to surgery, surgical approach and femoral offset.

**Conclusion:**

Hip hemiarthroplasty remains the gold standard for elderly patients with intracapsular neck of femur fractures. In each individual case the factors most strongly associated with postoperative dislocation should be recognised. Delays to surgery should be minimised and the posterior approach avoided. In addition to good surgical technique, particular attention should be paid to restoring the patient’s native femoral offset and post operatively those with cognitive impairment should be closely monitored.

## 1 INTRODUCTION

There were over 64,000 neck of femur fractures in the UK in 2013 and this number is set to rise as a result of an ageing population [1]. The majority (59%) of these patients sustain displaced intracapsular fractures, of which 80% are treated with a cemented hemiarthroplasty. This is in line with the current National Institute for Health and Clinical Excellence (NICE) guidelines [2]. The use of internal fixation, bipolar hemiarthroplasty and uncemented press-fit hemiarthoplasty has declined as the clinical evidence for cemented hemiarthoplasty has emerged. Total hip replacement is also an option for a subgroup of younger fit and independent patients. Nineteen percent of hip fractures were treated with total hip replacement in 2013.

Patients undergoing hip fracture surgery are often frail with multiple comorbidities. Mortality after one year is approximately 30% [3]. Medical complications are also common in the post-operative period. The official figure from the National Hip Fracture Database (NHFD) for re-operation for any cause is 1%, but such complications are often poorly reported [1]. The real rate of reoperation probably exceeds this. Specific indications for return to theatre are poorly recorded. Wound infection and dislocation are the most common in the early post-operative period. Revision to total hip replacement is required in the medium to long term for a small minority of patients.

Biomechanically, a hip hemiarthroplasty is inherently stable given its large head to neck ratio. For the femoral head to dislocate from the acetabulum it needs to displace by at least half the diameter of the head (jump distance). With a small-headed total hip replacement this distance may be only 11-14 mm. This figure increases to 20-25mm with the larger head diameter of a hemiarthroplasty (*e.g.* 50mm). Nevertheless, dislocations do occur. The literature reports an incidence of 1 to 15% [4-6]. Factors which may influence dislocation can be divided into 3 categories: patient factors, surgeon factors and surgical factors Table (**1**). Manipulation of some of these factors has the potential to reduce the incidence of dislocations and improve patients’ overall outcomes. Periprosthetic infection should be considered in all cases of dislocation. The American Association of Orthopaedic Surgeons (AAOS) recommends erythrocyte sedimentation rate (ESR) and C-reactive protein (CRP) as baseline investigations with selective use of aspiration and biopsy for patients with a higher probability of infection [7].

This article reviews the literature to provide a better understanding of the risk factors for hemiarthroplasty dislocation. A comprehensive review of the literature was performed using the search engine PubMed with the keywords ‘hip’, ‘hemiarthroplasty’ and ‘dislocation’. Two hundred and forty three articles were identified and assessed by the 3 authors independently. Data from fifty-two articles pertinent to the review on hemiarthroplasty dislocation epidemiology, risk factors and management were extracted in a standardised fashion (Table **2**).

## 2 PATIENT FACTORS

### 2.1 Mental Impairment and Neurological Conditions

The rehabilitation of hip fracture patients is a challenging process and involves the skills of a multidisciplinary team. Patients with mental impairment and/or neurological conditions are even more difficult to rehabilitate. They may also find it more difficult to comply with the usual hip-surgery precautions. The poor muscle control of patients with Parkinson’s disease or weakness from prior cerebrovascular accident give rise to particular concern. The literature reports high dislocation rates for such patients with some older papers reporting dislocation rates up to 37% [8]. More recent studies show lower rates which may reflect an improvement in both surgical techniques and post-operative rehabilitation for these patients. Ninh *et al* found a strong association (54.5% of dislocated versus 18.8% of non-dislocated HAs) between mental impairment and dislocation rates. The difference between the two groups was even more pronounced 12 months post operatively [9].

Such findings were not substantiated in a large single centre study analysing the outcomes of 3,525 patients over a 11 year period who underwent HA. In the group of patients who dislocated 11% had Parkinson’s disease versus 4.4% in the uncomplicated group [10]. Similarly, Staeheli only reported 1 dislocation in a series of 49 patients suffering from Parkinson’s disease [11].

## 3 SURGEON FACTORS

### 3.1 Surgical Experience

Several studies have incorporated surgeon seniority into the analysis of their results. Despite common sense suggesting that the dislocation rate would be higher with a junior surgeon, this has not been borne out in the published literature. Ames *et al* . found that case volume rather than surgeon grade was the most important factor in affecting dislocation rates while Enocson found no correlation between the two in a study of 720 cases [12, 13]. Unwin *et al* only found increased rates of dislocation with junior surgeons when hemiarthroplasty was performed using the posterior approach [14]. It is important to recognise that this paper dates from a time when capsular and external rotator repair was not a routine part of wound closure after a posterior approach. In a study analysing 3,525 intra-capsular neck of femur fractures over 11 years Salem *et al* found no difference in dislocation rates based on surgeon grade. This was thought to reflect the constant supervision from a senior surgeon [10].

Irrespective of surgical experience, emphasis should be placed on meticulous surgical technique with particular attention paid to implant positioning and soft tissue tensioning.

## 4 SURGICAL FACTORS

### 4.1 Implant Fixation

Current guidance advocates use of a cemented hemiarthroplasty implant. This is because of the lower risk of postoperative thigh pain, better early function and overall long term lower mortality rate [1, 15]. Polymethylmethacrylate (PMMA) acts as a grout between the prosthesis and bone providing it with immediate stability. As a result patients have less pain when compared to uncemented implants. Modern uncemented implants rely on osseous integration, whereas older designs were simply press-fit, relying upon the straight stem achieving a 3-point fix within the curved medullary cavity of the femur. One major drawback related to the use of cement is the rare but serious complication of bone cement implantation syndrome (BCIS) [16]. This can result in cardiac arrhythmias and cardiopulmonary collapse which can ultimately be fatal and thus should be avoided in frail patients considered too high risk to justify a cemented prosthesis. Steps to reduce the likelihood of BCIS include maintenance of adequate circulating volume and arterial pressure as well as close monitoring of end-tidal carbon dioxide during surgery. Careful preparation and drying of the femur and avoidance of cement pressurisation will further help to limit the incidence of BCIS in frail patients.

In a recent paper by Langslet *et al*. 112 cemented hemiarthroplasties were compared with 108 uncemented hydroxyapatite coated hemiarthroplasties in a randomised control trial (RCT) [17]. The femoral stems were sourced from different manufacturers but were coupled with the same bipolar head. Although significant differences were found in terms of function and peri-operative fractures, no dislocations were noted in either group. Deangelis *et al* performed a similar RCT comparing cemented and uncemented implants and also noted no dislocations for the duration of the trial [18].

Similar outcomes have been reported in other studies including: Figved *et al.* performed a RCT showing no difference in dislocation rates between the 2 prosthetic designs over a 5 year period [19]; Weinrauch *et al.* reviewed 1118 cases and found no significant difference when comparing uncemented Austin Moore implants to cemented Thompson implants over a 6 year period [20].

Varley *et al.* reviewed 81 papers and pooled data relating to hemiarthroplasty dislocation following the use of cemented and uncemented stems [21]. They reported dislocations in 144 of the 6,863 uncemented cases in comparison to 157 dislocations of the 4,322 cemented cases. This difference was not statistically significant when adjusted for surgical approach.

### 42 Unipolar Versus Bipolar Articulations

A bipolar prosthetic femoral head has 2 separate articulations Fig. (**1**). The large diameter outer head articulates with the native acetabulum. The smaller internal head sits within this and movement occurs between the two. This design was proposed to reduce acetabular wear, allow a greater range of hip movement and increase stability. Such articulations are more expensive (approximately £150 in our institution) than their unipolar counterparts [22]. They were used to try and reduce the incidence of acetabular pain requiring revision of a unipolar hemiarthroplasty to total hip replacement. Radiological evaluation of bipolar components used for neck of femur fractures has, however, revealed minimal movement between the inner and outer heads with the majority of movement being at the out shell/cartilage interface [23, 24]. Consequently, the Cochrane review from 2010 concluded that there was no evidence to support the use of the more expensive bipolar articulation [25].

Enocson *et al*. compared one of the largest consecutive series of 427 unipolar and 403 bipolar Exeter hemiarthroplasties and found no significant difference in dislocations rates (2.8 vs 3.0%). All operations were performed through an anterolateral approach and used the same stem (cemented Exeter) [26]. Furthermore, an earlier RCT from the same department analysing 120 patients found a similar overall complication rate and failed to show any functional advantage of one prosthetic head type over the other [27].

Other smaller studies have drawn the same conclusion with regard to dislocations: these include Calder *et al* (250 patients), Davison *et al.* (187 patients), Raia *et al* (115 patients), Ong *et al* (281 patients) and finally Paton (171 patients) [28-32]. These studies were less robust since the hemiarthroplasties were performed using different stems and some (Paton) also utilized different surgical approaches. The conclusions of these papers must therefore be interpreted with caution.

Kanto *et al* undertook a randomised control trial of 88 unipolar and 87 bipolar HAs. At 5-year follow-up they found a significantly higher dislocation rate in the unipolar group. Both groups used the same stem inserted through a posterior surgical approach and patients underwent the same postoperative rehabilitation. The authors did not discuss possible reasons for these results which contrast with the earlier literature but did find that all-cause revision rates for the 2 groups were equal [33].

### 4.3 Posterior Versus Lateral Approach

Although a trans-trochanteric approach allows the best exposure to the hip joint it is invasive and inappropriate for HA. Thus, the two most commonly used surgical approaches in HA surgery are the anterolateral and posterior approaches. Theoretically, the anterolateral approach affords greater stability since the orientation of the acetabulum favours posterior dislocation. However, this approach may damage the gluteus medius and/or its nerve supply. The resultant Trendelenberg gait is disabling and very difficult to treat. The posterior approach is a muscle sparing-approach. Specifically there is no dissection through, or risk of nerve damage to, the hip abductors. The posterior approach also offers better exposure of the femoral canal, allowing easier passage of reamers and rasps. This may reduce the incidence of intra-operative calcar fractures.

One of the earliest published RCT on dislocations of hip hemiarthroplasty concluded that the anterolateral approach was the safest approach when performing HA in patients with a femoral neck fracture. However, their findings were based on a higher mortality rate and not differences in dislocations *per se* [34]. The validity of their results was questioned by Parker *et al* in a Cochrane Review as the study had poor methodology [35].

Eight years later, Paton *et al.* assessed 171 neck of femur patients retrospectively primarily to ascertain if bipolar devices offered greater stability than traditional unipolar devices [32]. The study failed to show any difference between the two types of prosthetic head but did favour the lateral approach. Close scrutiny shows, however, only a trend towards fewer dislocations with the lateral approach. The results did not reach statistical significance (p<0.08).

A larger prospective study involving 531 patients reported an increased dislocation rate when the posterior approach was used [36]. Although dislocation rates were lower in the anterolateral group the other associated complications (*e.g.* Trendelenberg gait) led the authors to conclude that surgeons should use the surgical approach with which they are most familiar.

Many subsequent studies have favoured the anterolateral approach over the posterior approach basing their recommendations primarily on the lower dislocation rate. These studies include that of Unwin *et al* who analysed 3118 consecutive HAs and noted a 3.3% versus 9% dislocation rate for anterolateral versus posterior approach respectively [14]. They found that even repair of the short external rotators did not confer sufficient stability to make the posterior approach safe. Pajarinen *et al* . noted the most significant independent factor predisposing to dislocation was the use of the posterior approach (16%) [37]. Bush *et al* found no dislocations with an anterolateral approach (p<0.0033) [38]. Biber *et al* analysed 704 consecutive patients and found statistically significant fewer dislocations with a transgluteal approach (0.5% versus 3.9%) [39]. Abram *et al* reviewed 807 Thompson HAs with figures for dislocation with the posterior approach of 13% compared with 2.1% when an anterolateral approach was used [40]. Enocson showed that in a cohort of 739 patients the posterior approach was the sole factor associated with a significant increase in dislocation rates [12].

Large studies that have reviewed and re-analysed data from smaller cohorts have also found a higher dislocation rate when the posterior surgical approach is chosen for hip hemiarthroplasty. Hip Fracture Registry data adds further support to this finding. In 2004 a comprehensive review of the literature by Varley and Parker demonstrated a higher dislocation rate using the posterior approach. With pooled data these authors demonstrated that the rate of dislocation when a posterior approach was used was more than twice that seen after surgery through an anterolateral surgical exposure (5.1% as opposed to 2.4%) [41].

Rogmark *et al* analysed 33,205 procedures in the Norwegian and Swedish National Hip Fracture Registries and demonstrated that the posterior approach clearly increased the risk of re-operation due to dislocation [42].

There is, however, also some evidence in the literature demonstrating no significant increase in dislocation rates with the posterior approach. Perhaps the best of the studies demonstrating no difference in dislocation rates in respect of the surgical approach is that of Sierra *et al* . They undertook a comprehensive, but retrospective, study evaluating the incidence and contributing factors in dislocations of bipolar hip hemiarthroplasties [43]. The study was based at a single institution and included 1812 patient episodes over a 27 year period. The authors concluded that the cumulative probability of dislocation at 1 year, 5 years, 10 years and 20 years was 1.1% and that there was no significant association with the surgical approach.

It is worth noting that there is no literature showing a lower dislocation rate with the posterior approach. Also, only 3 of these studies commented on whether repair of the short external rotators and capsule was performed. Of these, only two studies (Enocson and Abram) formally assessed the impact of the posterior repair on dislocation rates which did not reach statistical significance [12, 40].

### 4.4 Capsular Repair *Vs* Capsulectomy

Although there is good reason to support capsular repair when performing a hemiarthroplasty or a total hip arthroplasty using a posterior surgical approach, there is no published literature to support capsular repair over capsulectomy following surgery **via** an anterolateral approach [44, 45]. Using cadaveric specimens, Hughes *et al* recently investigated the contribution of the anterior capsule to the stability of a cemented HA performed using an anterolateral approach [46]. They found a statistically significant difference in the peak torque force required to dislocate a HA depending on whether it had undergone a capsular repair or not (22.96Nm vs 5.56Nm). The authors concluded that repair of the capsule may contribute to stability of a HA performed using the anterolateral approach. Further studies will be necessary to assess the validity of this finding in the clinical setting.

### 4.5 Type of Stem

Very few studies have focussed solely on the type of stem used for hip hemiarthroplasty in relation to dislocation rates. Bidwai *et al* undertook a prospective audit of patient outcomes after a change of practice from using the Thompson stem (766 patients) to the Exeter trauma stem (388 patients) in a single centre study [47]. There was no difference in the rate of dislocation. Similarly there was no difference in the ratings given to the postoperative radiographs. The one positive conclusion from this study was the unsubstantiated conclusion that the ETS may be easier to revise to a total hip replacement.

## 5 OTHER FACTORS

### 5.1 Previous Failed Surgery

Secondary HA after failed internal fixation is a technically more difficult operation. Often the patient has poor quality soft tissues owing to previous surgeries and poor muscle function prior to the injury. Roberts *et al* and Enocson *et al* found marked increased dislocation rates when looking at delayed hemiarthroplasty performed for failed internal fixation [26, 48]. Both studies found a higher dislocation rate compared to control groups who had a hemiarthroplasty primarily (4% vs 0.8%; 6.5% vs 2.1%). This contrasted with an earlier cohort study of 739 patients by Enocson which showed no significant difference between primary and secondary HAs. This study has confounding variables, however, since neither surgical approach nor implant was standardised. Patients had surgery through either the anterolateral or posterior approach, and the study included 3 different stems [12].

### 5.2 Delay to Surgery

Current guidelines for hip fracture surgery in the UK recommend operative treatment within 36 hours [2]. This is mostly founded on the increased morbidity and mortality figures for patients operated on beyond this times. There is also some evidence, however, to suggest that even a shorter delay to surgery of 24 hours can have a detrimental effect on HA dislocation rates. Salem *et al* reported a fourfold increase in dislocations following a 24 hour delay to surgery with a further dramatic increase to tenfold at 36 hours [10]. This was thought to reflect increased soft tissue swelling and consequent compromise to surgical exposure and closure.

Madanat *et al.* also found a significant dislocation risk with a delay to surgery of over 48 hours. Their study also showed a propensity for dislocations to be secondary to falls (59%) which they linked to the deterioration in the patients’ physical ability that resulted from delayed surgery [49].

Whilst delay to surgery may have a direct influence upon the incidence of subsequent dislocation, it must be borne in mind that prior to the introduction of specific guidance to expedite hip fracture surgery it was the more frail patients who were delayed to allow time to ‘optimise them for anaesthesia.

### 5.3 Radiographic/Anatomical Factors

Femoral offset, residual neck length, and anatomical factors indicative of hip dysplasia such as the centre edge angle of Wiberg (CEA) and acetabular index will also play a role in implant stability. Femoral neck offset is defined as the perpendicular distance between the intramedullary axis of the femur and the centre of rotation of the native or prosthetic femoral head. This may be affected intraoperatively by changing the version or the varus/valgus angulation of the stem as well as by choice of prosthesis. The CEA is the angle measured between a vertical line drawn through the centre of the femoral head and a line drawn from the centre of the head to the lateral aspect of the acetabulum. Residual neck length is the retained length of host femoral neck from the femoral cut to the top of lesser trochanter. Acetabular index is defined as the angle between a horizontal line parallel to the tear drop-ischial line and the roof of the acetabulum Figs. (**2** and **3**). Ninh *et al* conducted a retrospective study analysing the effects of such factors, amongst others, in 144 patients who were all followed up at 1 year post cemented HA [9]. Eleven dislocations were noted during this time and their radiographic data was compared to 83 random patients who did not dislocate. The authors divided their findings in early (6 weeks) and late (12 months) and found isolated contralateral femoral offset and CEA to be a significant factor at both stages of assessment with the patients suffering dislocation having higher offset in their contralateral hip and a smaller CEA on the prosthetic side. Isolated femoral neck offset was only significant at 12 months once again with those suffering dislocation having a lower offset. Residual femoral neck and acetabular index were not significant at either stage. Only the CEA remained significant when combined with clinical factors such as age, surgical approach and mental impairment. A decreased CEA was also noted to be more common in males giving them a four times higher dislocation rate overall.

In a similar study Madanat *et al.* found that a smaller centre edge angle (CEA) and decreased femoral offset (FO) resulted in higher dislocation rates. However, in contrast to the Ninh study, they also found a shorter residual femoral neck to be significant (13mm versus 16mm, P = 0.029) [49]. The study used a single type of prosthesis and a single standardised approach (posterolateral) which may have limited confounding variables. The clinical significance of this is questionable given that there was no control for the length of the native femoral neck prior to resection, the size of patient or the measurement error on plain radiographs.

## 6 DISCUSSION

Dislocation of a hip hemiarthroplasty is a rare but potentially devastating complication. The six-month mortality following a single episode of dislocation is 65% at 6 months, rising to 75% if a second dislocation occurs [50]. There is also a cost implication. de Palma *et al* reported an increased cost of 472% over a baseline uncomplicated HA [51]. Interestingly, the same study found the treatment of a HA dislocation to be more costly than that of either a dislocated primary total hip replacement or revision total hip replacement. This almost certainly reflects the longer length of hospital stay required for the frail hip fracture patients.

The majority of reported dislocations occur within in the first month following surgery [10, 12, 40, 49, 52]. Initial treatment entails closed reduction but re-dislocation rates are high. In a series of 8631 HAs Salem reported closed reduction to be definitive treatment in only 23% of cases [10]. A similar figure of 30% is quoted by Sierra *et al* although this related solely to bipolar hemiarthroplasties which are more difficult to reduce closed due to the double articulation [43]. In 2 separate papers Enocson reported second dislocation rates of 33% and 78% respectively (unipolar vs bipolar heads) after successful primary reduction with a large majority of these (9/14) requiring repeated closed reductions in the latter series [12, 26].

There are no hard and fast predictors of dislocation in hemiarthroplasty surgery. There are, however, certain factors which stand out in the literature. Recognition of these will help to reduce the rate of dislocation. Delay to surgery should be strongly avoided with surgery being prioritised and undertaken on the day of, or day after admission as recommended by the NICE guidelines [2]. Although this may prove difficult both because of limited theatre availability and the need for preoperative patient optimisation, a multidisciplinary team assessment and reorganisation of theatre facilities has been shown to produce significant clinical and financial benefits [1]. Intraoperatively, the posterior approach should be avoided and, in addition to meticulous surgical technique, attention should be paid to maintaining the patient’s native offset where possible. Postoperatively patients with impaired cognitive function should be monitored closely to try and encourage the usual hip-surgery precautions.

Even when all these factors are understood, dislocations will still occasionally occur. An initial attempt at closed reduction should be made. Patients should be warned of the possibility of further dislocations and the need for revision surgery. Bipolar articulations are more difficult to reduce than unipolar articulations so the need for open reduction is more likely. Similarly, if radiographs demonstrate suboptimal implant positioning or acetabular dysplasia (decreased CEA), open revision may be necessary. Revision options include conversion to total hip replacement or excision arthroplasty. This decision will clearly depend on the patient’s mental state, premorbid mobility and independence and their physiological reserve.

## CONCLUSION

Hip hemiarthroplasty remains the gold standard operation for elderly patients suffering an intra-capsular hip fracture. With the changing population demographics an increasing number of such operations will be required each year. Dislocation is a major complication. It requires reoperation with associated morbidity and mortality. Keeping dislocation rates to a minimum is therefore a matter of high importance in orthopaedic trauma practice. There is good evidence that a bipolar hemiarthroplasty is more difficult to reduce than a unipolar design with no good evidence of advantages (acetabular erosion and stability). Unipolar heads are therefore preferred. Cemented stems are preferred for all but the frailest patients with regard to overall complications (cement implantation syndrome the one controversial exception) and there is no evidence that a cemented stem affects dislocation rate.

There has been considerable focus on the incidence of dislocation with different surgical approaches. Much of the literature is confused, with too many variables. However, the good quality papers that do exist, and the reports and reviews that analyse pooled data, provide good evidence that there is a greater risk of postoperative dislocation when a posterior surgical approach is used. This probably remains true even with modern closure techniques.

Further reduction in dislocation rates might be achieved if the same attention that is given to implant positioning in total hip arthroplasty is extended to the hemiarthroplasty arena. There is presently no published literature that explores this topic.

## Figures and Tables

**Fig. (1):**
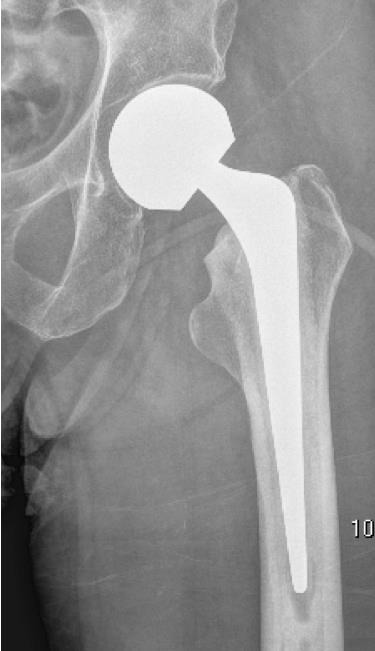
Picture of a bipolar prosthetic head demonstrating the 2 separate articulations.

**Fig. (2):**
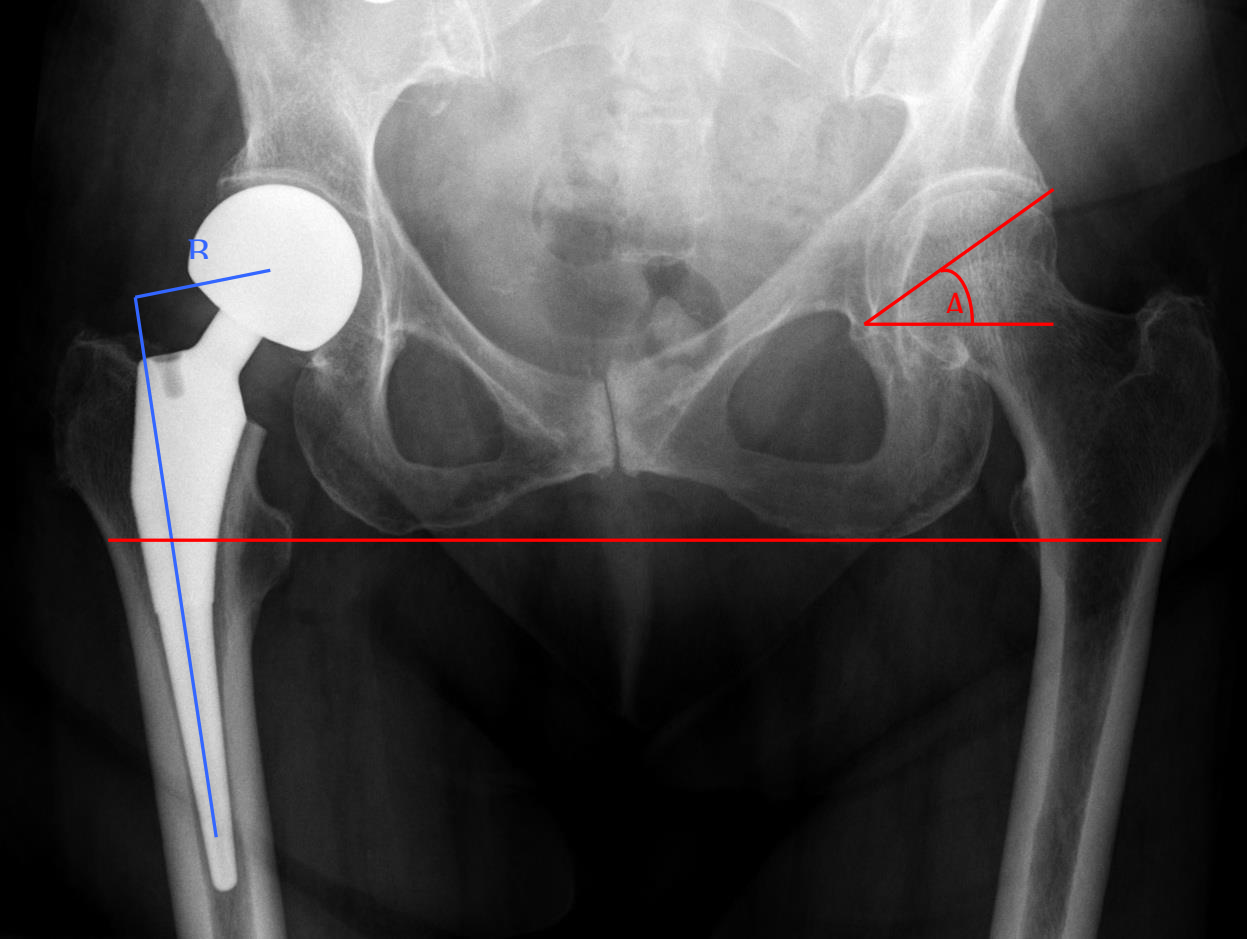
Radiograph of an uncemented hemiarthroplasty with a unipolar head showing the acetabular index (A - red) and femoral neck offset (B - blue).

**Fig. (3):**
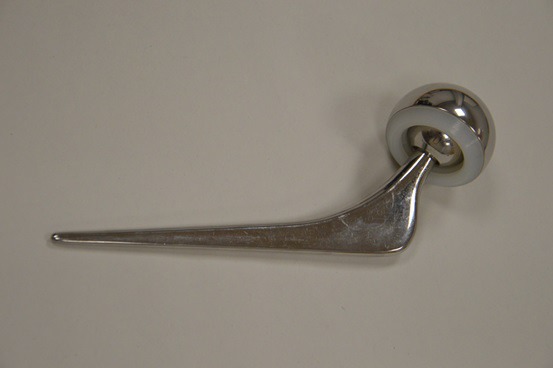
Radiograph of a cemented hemiarthroplasty with a unipolar articulation demonstrating centre edge angle (yellow) and residual femoral neck length (green).

**Table 1:** Factors affecting risk of dislocation following a hip hemiarthroplasty.

Patient	Surgeon	Surgical
Neurological impairment (stroke)	Surgeon volume (not seniority)	Time to surgery – theatre availability
Muscle weakness (Parkinson’s)	Soft tissue tensioning	Surgical approach
Previous failed internal fixation of hip	Implant positioning	Capsular repair
Hip dysplasia (decreased CEA/offset)	-	-
Male gender (related to CEA)	-	-
Time to surgery – need for preoperative optimisation	-	-

**Table 2:** Summary of some of the more relevant papers in HA dislocation.

** Study Author **	** Origin / Year **	** Study Population **	** Outcome and results **
**PATIENT FACTORS** **Mental Impairment and Neurological conditions**			
Coughlin *et al* [8]	Canada, 1980	49 HAs in 47 patients with Parkinson’s disease/stroke	37% dislocation rate
Ninh *et al* [9]	USA, 2009	144 patients undergoing HA at 1 year post op	54% dislocation rate with mental impairment
Salem *et al* [10]	UK, 2014	3,525 HAs over 11 years	No correlation with mental impairment
Staeheli *et al* [11]	USA, 1988	49 patients with Parkinson’s disease undergoing HA	1% dislocation rate
**SURGEON FACTORS** **Surgical experience**			
Enocson *et al* [12]	Sweden, 2008	720 HAs in 739 patients	No correlation between grades and dislocation
Unwin *et al* [13]	UK, 1994	2906 patients undergoing HA	Increased dislocation for junior grade only when using posterior approach
Salem *et al* [10]	UK, 2014	3,525 HAs over 11 years	No correlation between grades and dislocation
**SURGICAL FACTORS** **Implant fixation**			
Langslet *et al* [17]	Norway, 2014	RCT: 112 cemented vs 108 uncemented	No correlation between fixation and dislocation
Deangelis *et al* [18]	USA, 2012	RCT: 274 HAs in 269 patients	No correlation between fixation and dislocation
Figved *et al* [19]	Norway, 2009	RCT: 112 cemented vs 108 over 5 years	No correlation between fixation and dislocation
Weinrauch *et al* [20]	Australia, 2006	1118 Austin Moore vs Thompson over 6 years	No correlation between fixation and dislocation
Varley *et al* [21]	UK, 2004	81 papers reviewed – 6,863 uncemented vs 4,322 cemented	No correlation between fixation and dislocation
**Unipolar vs Bipolar**			
Enocson *et al* [26]	Sweden, 2012	427 unipolar vs 403 bipolar	No correlation between articulation and dislocation
Calder *et al* [28]	UK, 1996	RCT: 118 Monk vs 132 Thompson HAs	No correlation between articulation and dislocation
Davison *et al* [29]	UK, 2001	RCT: 90 Thompson vs 97 Monk HAs	No correlation between articulation and dislocation
Raia *et al* [30]	USA, 2003	RCT: 60 unipolar vs 55 bipolar	No correlation between articulation and dislocation
Ong *et al* [31]	USA, 2002	101 bipolar vs 180 unipolar HAs	No correlation between articulation and dislocation
Paton *et al* [32]	UK, 1989	108 unipolar vs 63 bipolar HAs	No correlation between articulation and dislocation
Kanto *et al* [33]	Finland, 2014	88 unipolar vs 87 bipolar RCT at 5 year follow up	Significant unipolar dislocation rate
**Anterolateral vs posterior approach**			
Paton *et al* [32]	UK, 1989	78 lateral vs 93 posterior HAs	Not statistically significant
Keene *et al* [36]	UK, 1993	302 anterolateral vs 229 posterior HAs	Increased dislocation with posterior approach
Unwin *et al* [13]	UK, 1994	2150 anterolateral vs 1656 posterior HAs	Increased dislocation with posterior approach – 3.3 vs 9%
Pajarinen *et al* [37]	Finland, 2003	338 patients undergoing HA	Increased dislocation with posterior approach
Bush *et al* [38]	USA, 2007	375 patients undergoing HA	Increased dislocation with posterior approach – 0 vs 4.5%
Biber *et al* [39]	Germany, 2012	217 anterolateral vs 487 posterior HAs	Increased dislocation with posterior approach – 0.5 vs 3.9%
Abram *et al* [40]	UK, 2015	753 anterolateral vs 54 posterior HAs	Increased dislocation with posterior approach – 2.1 vs 13%
Enocson *et al* [12]	Sweden, 2008	431 anterolateral vs 305 posterior HAs	Increased dislocation with posterior approach – 3% vs 8.5/13% (repair/no repair)
Varley *et al* [41]	UK, 2004	84 papers reviewed – 6,026 anterolateral vs 7,912 posterior HAs	Increased dislocation with posterior approach – 2.4 vs 5.1%
Rogmark *et al* [42]	Sweden, 2014	21,206 anterolateral vs 11,999 posterior HAs	Increased dislocation with posterior approach
Sierra *et al* [43]	USA, 2006	1558 anterolateral/lateral vs 254 posterior HAs over 27 years	No difference in cumulative probabilities at 1, 5, 10 and 20 years
**Capsular repair vs capsulectomy**			
Hughes *et al* [46]	UK, 2015	Cadaveric study of 10 hips	Increased stability with capsular repair
**Stem type**			
Bidwai *et al* [47]	UK, 2012	766 Thompson vs 388 Exeter trauma stem	No difference between stem types
**OTHER FACTORS** **Previous failed surgery**			
Roberts *et al* [48]	UK, 2002	100 HA as revision procedure vs 730 primary HAs	Increased dislocation rate following previous failed surgery – 0.8 vs 4%
Enocson *et al* [12]	Sweden, 2008	720 HAs in 739 patients	No correlation between dislocation rate and previous failed surgery
**Delayed surgery**			
Salem *et al* [10]	UK, 2014	3,525 patients undergoing HA over 11 years	4-fold increase with 24 hours delay/10-fold with 36 hour delay
Madanat *et al* [50]	Finland, 2012	602 patients undergoing HA	Significant risk of dislocation over 48 hours
**Radiographical/anatomical factors**			
Ninh *et al* [9]	USA, 2009	144 patients undergoing HA at 1 year post op	Higher dislocations rate with decreased femoral offset and CEA
Madanat *et al* [50]	Finland, 2012	602 patients undergoing HA	Higher dislocation rate with decreased femoral offset and decreased CEA

## LIST OF ABBREVIATIONS

AAOS: = American Association of Orthopaedic Surgeons
BCIS: = Bone cement implantation syndrome
BPT: = Best practice tariff
CEA: = Centre edge angle
CRP: = C reactive protein
ESR: = Erythrocyte sedimentation rate
FO: = Femoral offset
HA: = Hemiarthroplasty
NHFD: = National Hip Fracture Database
NICE: = National Institute for Clinical Excellence
RCT: = Randomised control trial
